# Infants and Mobiles: Developing an Understanding of Cause and Effect

**DOI:** 10.1111/desc.70211

**Published:** 2026-05-13

**Authors:** Xia Xu, Jochen Triesch

**Affiliations:** ^1^ Frankfurt Institute for Advanced Studies, Frankfurt am Main, Germany; ^2^ Xidian‐FIAS International Joint Research Center Xi'an China; ^3^ Goethe University, Frankfurt am Main, Germany

## Abstract

In the mobile conjugate reinforcement paradigm, an infant's leg is connected to a mobile via a string, allowing the infant to move the mobile via moving their leg. Over a few minutes, infants exhibit an increase in the frequency of movement of the connected leg. This behavior is sometimes interpreted as an indication that infants experience the efficacy of causal control. However, some researchers have argued that an underlying causal model is not necessary and that a simple reinforcement model that favors mobile movements can explain this behavioral pattern. Interestingly, after the mobile is disconnected from the leg, some infants transiently show an even higher frequency of movement, a phenomenon known as the extinction burst, that is hard to reconcile with a simple reinforcement learning model alone. In this study, we propose different computational models and study to what extent they are capable of capturing infants' behavior. In particular, we construct an active‐learning causal model that is capable of discovering the underlying cause‐effect relationship on the fly without the need to specify either the cause or the effect in advance. We also propose an active‐learning mechanism based on expectation violation, that can be combined with the proposed causal model and a number of alternative models, including a naïve reinforcement model, to give rise to an extinction burst. Overall, our work sheds light on possible learning mechanisms giving rise to infant's developing understanding of cause and effect relationships.

**Summary:**

- A causality‐driven model is proposed that successfully simulates infant behavior in the mobile paradigm.
- The causal model actively discovers the underlying causal mechanism, while alternative models fail under specific simulated conditions.
- An active‐learning mechanism based on expectation violation is introduced that unifies causal learning and hypothesis‐testing within a single, coherent framework.
- We show that this mechanism successfully models the extinction burst and demonstrates robustness across different experimental settings.

## 1 Introduction

The development of an infant's sense of agency—the recognition that their actions can influence external events—has been extensively explored through studies examining increased action frequency in response to contingent effects. Research rooted in operant conditioning paradigms, such as Rovee–Collier's mobile conjugate reinforcement experiments (Rovee and Rovee 1969), demonstrates that infants as young as 2–3 months old increase leg‐kicking when such actions reliably produce movement in an overhead mobile, highlighting early sensitivity to action‐outcome contingencies. Similarly, Watson (1972) observed that infants exhibit heightened limb movements when paired with auditory or visual feedback, suggesting that even neonates engage in “contingency learning,” wherein predictable reinforcement strengthens action repetition. Advances in methodology, including neurophysiological measures, have further elucidated how infants' brain activity correlates with agency experiences, particularly when actions like vocalizations or gestures elicit caregiver responses (Hauf and Prinz 2004). Such findings underscore the role of reciprocal social interactions in refining agency, as infants amplify actions that provoke reliable external feedback (Bakeman et al. 1996). However, variability in developmental trajectories highlights the interplay of intrinsic factors (e.g., attentional capacity) and environmental responsiveness. Collectively, this body of work illustrates that the amplification of action frequency in the presence of contingent effects serves as a foundational mechanism through which infants refine their understanding of causality and volition, marking critical milestones in early cognitive development.

While the amplification of action frequency in response to contingent effects suggests infants are developing a sense of agency, a critical limitation of existing computational accounts, such as those based on simple reinforcement mechanism, is their reliance on pre‐specified cause‐and‐effect pairings. Proponents of associative learning argue that pre‐wired mechanisms are sufficient, a view supported by “babybot” simulations (Zaadnoordijk et al. 2018) where behaviors like kicking increase simply because they are reinforced by a pre‐defined reward, for example, for mobile movement. However, this approach is not ecologically viable in open‐ended environments where infants must first autonomously discover which of their many potential actions might cause an effect amidst a backdrop of spurious statistical correlations. These models bypass the fundamental cognitive challenge of identifying a true contingency from a vast space of possibilities, as they begin with the causal link already defined as a reward pathway. This highlights a crucial gap: the need for a mechanism that captures the process of causal discovery on the fly, mirroring an infant's active, hypothesis‐testing exploration of their environment without a priori knowledge of what is controllable.

Separately, the extinction burst phenomenon—a temporary, sharp increase in action frequency when an expected effect is abruptly removed—presents a significant challenge to operant conditioning models based on reinforcement mechanisms. In the mobile paradigm, after the acquisition phase, infants often kick with even greater frequency immediately following the disconnection of the mobile. This behavior is difficult to reconcile with simple reinforcement mechanism, where the removal of reinforcement should lead to a monotonic decay in action probability. While “babybot” simulations can replicate the general increase in limb‐specific action, they fail to provide a compelling mechanism for this transient surge, which is often interpreted as an infant's attempt to regain control over a disrupted contingency. This discrepancy suggests that the extinction burst is not a simple artifact of reinforcement history but may reflect a more sophisticated cognitive process (Bednarski et al. 2022), pointing to the need for a more advanced mechanism capable of explaining this distinct behavioral signature.

To address these twin challenges, this study proposes a causality‐driven model that autonomously discovers cause‐effect relationships on the fly, freeing it from the need for pre‐wired reward functions. The model formalizes causal inference by quantifying the distributional divergence between action‐contingent and baseline outcomes, enabling it to learn robustly in complex scenarios where alternative models fail. Specifically, it successfully infers causality in an “inverted case,” where an action suppresses an outcome, and in a “bimodal case,” where an action increases outcome entropy—conditions where a reinforcement model and a controllability‐based model fail to capture the cause‐effect relationships. Furthermore, the model introduces a novel “surprise” term that computationally operationalizes expectation violation. This mechanism, inspired by a neuro‐cognitive finding (Zaadnoordijk et al. 2020), explains the extinction burst as a quantifiable prediction error that triggers a compensatory increase in exploratory action, thereby unifying causal learning and hypothesis‐testing within a single, coherent framework.

### 1.1 Direct Reinforcement and Causal Models: The Necessity of Autonomous Discovery in Open‐Ended Learning

The debate over the necessity of explicit causal models to explain learning in the mobile paradigm hinges on a fundamental distinction between two computational problems: policy optimization versus causal discovery. Proponents of simple reinforcement models posit that the infant's behavior can be explained as an optimization process. In this view, supported by “babybot” simulations (Zaadnoordijk et al. 2018), the mobile's movement acts as a pre‐defined reward signal within a fixed reward function. The model's task is one of simple reward maximization: it learns a policy to increase the frequency of actions that produce this reward, without statistically forming an explicit representation of the underlying cause‐and‐effect mechanism. The critical limitation of this approach is its reliance on a pre‐specified reward function, which effectively hard‐codes the causal link it purports to explain. This formulation fails to address the true ecological challenge faced by an infant: the problem of credit assignment in an open‐ended environment. Infants are not provided with a reward oracle; they must first autonomously discover which of their many potential actions (e.g., kicking, vocalizing, and gazing) is causally responsible for which potential environmental effect (e.g., mobile motion and caregiver response) from a vast space of spurious statistical correlations. In contrast, causal models are designed to solve this discovery problem. They actively infer these relationships through *exploratory intervention*, mirroring the hypothesis‐testing behavior observed in infants (Begus and Bonawitz 2024). Arguably, this capacity to build a causal model of the world on the fly, rather than merely optimizing a policy for a pre‐defined objective, is essential for capturing the generative and robust nature of early cognitive development.

Building on Judea Pearl's causal framework (Pearl 2009), which emphasizes interventions as critical components of causal reasoning, we propose a computational model that integrates active exploration with a causal discovery mechanism to simulate infant learning in the mobile paradigm. We term the proposed causality measure the causal action influence score (CAIS). Direct reinforcement models, while effective at optimizing predefined action‐reward links, struggle to autonomously discover causal relationships in open‐ended environments where infants must infer which actions could produce any effects. Our model addresses this gap by formalizing causal discovery as an iterative process: the model infant actively intervenes on variables (e.g., limb movements) and computes a distance measure between baseline and conditional outcome probability distributions (e.g., mobile motion patterns) to quantify causal strength. The model dynamically updates hypotheses about action‐effect contingencies, mirroring infants' trial‐and‐error exploration. This approach bridges causal reasoning with statistical learning, offering a unified account of how agents might autonomously discover and test causal relationships without pre‐specified reward functions, aligning with experimental evidence for infants' agency‐driven exploration (Needham et al. 2017).

We also evaluate a controllability‐based model that identifies contingency via entropy reduction. We show that this method fails when actions alter the outcome's probability distribution without significantly reducing entropy. In contrast, our causal model successfully captures these contingencies by detecting distributional changes independent of entropy fluctuations.

### 1.2 Expectation‐Violation and Probability Density Estimation: Modelling Extinction Bursts Through Distributional Surprise

The phenomenon of extinction bursts can be modelled through the lens of expectation violation, a concept supported by an EEG study (Zaadnoordijk et al. 2020) on the emerging sense of agency in infants. In the study, 3‐ to 4.5‐month‐old infants learned that specific limb movements triggered audiovisual effects. When this established contingency was abruptly discontinued, infants who exhibited a neural signature of expectation violation—a mismatch negativity (MMN) component in their EEG—also displayed a limb‐specific extinction burst. The MMN, a known marker for detecting unexpected deviations from learned patterns (Garrido et al. 2009), appeared about 200–350 ms after movement onset, signaling that the infants' brains had registered the violation of the expected outcome. Importantly, a pronounced behavioral extinction burst was only demonstrated by the subgroup of infants who also showed the MMN response. This suggests that the burst originates from a mismatch between predicted and actual outcomes, not merely from pre‐wired associative reinforcement. This link between neural activity and behavior supports the hypothesis that extinction bursts are active attempts to restore or reassess an expected causal relationship, driven by the infant's developing causal models of agency.

To capture this dynamic within a computational model, the causal discovery mechanism is extended by an expectation violation mechanism, where low probability outcomes indicate high prediction errors. During the extinction phase, the absence of the expected outcome (e.g., mobile motion) causes the observed outcome's probability density to plummet when compared to the model's predicted probability distribution. This drop quantifiably represents the expectation violation. This density‐based metric, analogous to the MMN signal, triggers a compensatory mechanism within the model: it intensifies the actions historically linked to the now‐absent effect, thereby simulating the limb‐specific extinction burst. This approach effectively bridges causal reasoning with statistical expectation‐violation detection. It demonstrates that extinction bursts can emerge naturally as an agent attempts to resolve low‐probability events by reinforcing exploratory behaviors, which aligns with developmental theories of agency as an interplay between causal discovery and environmental engagement (Blanco and Sloutsky 2024).

## 2 Inferring Causality and Detecting Surprise

The concept of causality has been variably interpreted and operationalized across behavioral sciences (Sobel 1995). In this work, we adopt Judea Pearl's formal framework of causal inference (Pearl 2009), employing intervention‐based definitions through the do‐operator to analyze action‐outcome relationships. Consider a binary action, when there are no confounders involved between (random) variables of interest. The estimand of one variable after intervention on the other can be expressed either as:

(1) $$p\left(m|do\left(a\right)\right)=p\left(m|a\right)$$

when a causal link a→m is present between action a and outcome m, or:

(2) $$p\left(m|do\left(a\right)\right)=p\left(m\right)$$

when the two variables a and m are independent. The divergence between these two outcome distributions Equations 1 and 2 quantifies causal effects, measuring the degree to which an action systematically influences the probability of a specific outcome. Here we propose the CAIS using the Wasserstein‐2 distance as the distributional distance metric:

(3) $$C\left(m|a\right)=W_{2}\left(p\left(m|a\right),p\left(m\right)\right)\;.$$

During learning, the agent π improves its estimate of the CAIS for each possible action and uses it to guide the next action. The precise action selection procedure will be discussed in further detail in Section 3.

To compute the CAIS, we adopt quantile regression (Petersen and Müller 2019) to estimate the $W_{2}$ distance:

(4) $$W_{2}\left(p_{1},p_{2}\right)=\int_{0}^{1}{\left(q_{1}\left(\tau \right)-q_{2}\left(\tau \right)\right)}^{2}d\tau \;,$$

which calculates the Wasserstein‐2 distance (Vaserstein 1969) between probability distribution $p_{1}$ and $p_{2}$ using corresponding quantile functions $q_{1}$ and $q_{2}$ as a function of the quantile τ. We adopted a grid of 49 quantiles (2%–98%). While a denser grid provides finer resolution, a 2% increment is adequate for our modelling objectives. See Appendix A for the effect of different number of quantiles. To learn the quantile functions, we adopt the modified quantile Huber loss proposed in Dabney et al. (2018):

(5) $$L_{\kappa }\left(u\right)=\left\{\begin{array}{cc} \frac{1}{2}u^{2}, & \mathrm{if}\left|u\right|\leq \kappa \\ \kappa \left(\left|u\right|-\frac{1}{2}\kappa \right), & \mathrm{otherwise} \end{array}\right.\;,$$

(6) $$u\left(q\left(\tau \right),\hat{y}\right)=\left\{\begin{array}{cc} \tau \cdot \left(q\left(\tau \right)-\hat{y}\right), & \hat{y}\leq q\left(\tau \right)\\ \left(1-\tau \right)\cdot \left(\hat{y}-q\left(\tau \right)\right), & \hat{y}>q\left(\tau \right) \end{array}\right.\;,$$

where u is the quantile error of each quantile τ and its quantile value $q\left(\tau \right)$ with respect to the target value $\hat{y}$ which, in the mobile paradigm, refers to the resultant movement of the mobile. Throughout the paper we adopt κ=1 for simplicity.

The integration of Wasserstein distance with quantile regression offers distinct advantages over conventional Kullback–Leibler (KL) divergence‐based mutual information—a common method for quantifying distributional differences. First, unlike the KL divergence, which lacks metric properties, the Wasserstein distance satisfies symmetry and the triangle inequality, enabling geometrically meaningful comparisons of distributions. Second, quantile regression bypasses the computational burden of explicit probability density estimation, which often necessitates nested algorithms during training, by directly modelling quantiles of the outcome distribution. Third, this approach aligns with neurobiological insights: quantile regression's focus on reward distribution tails mirrors dopamine‐driven reward expectation mechanisms in the human brain (Dabney et al. 2020), potentially linking causal inference to neural processes governing stochastic reward‐seeking behaviors and learning. It is crucial, however, to interpret these advantages at the appropriate level of analysis. The use of this specific mathematical formalism is a convenient computational modelling choice, motivated by its descriptive power and efficiency in simulating the hypothesized psychological process. We do not claim that the infant brain performs quantile regression or computes optimal transport (Vaserstein 1969) in this way. Rather, the model posits a more abstract mechanism: that the infant's learning systems are sensitive to distributional shifts (Dabney et al. 2020). The Wasserstein‐quantile approach provides a robust and principled way to formalize and implement this abstract hypothesis.

To model the extinction burst effect, inspired by the expectation violation in the form of the MMN observed in EEG data, we define a measure of surprise. For this we consider the outcome distributional quantile interval as an inverse measure of probability density, where lower‐density regions (sparse probability mass) correspond to wider quantile intervals, reflecting greater surprise:

(7) $$S_{t}\left(m_{t},a_{t}\right)=\left|q_{m_{t}}\left(\tau_{i+1}\right)-q_{m_{t}}\left(\tau_{i}\right)\right|\;,$$

where $a_{t}$ is the action that the agent is performing in the current time step t. $\left[q\left(\tau_{i}\right),q\left(\tau_{i+1}\right)\right]$ refers to the quantile interval into which the outcome $m_{i}$ falls. Notice that wider quantile intervals inherently reflect lower probability density and therefore higher surprise, enabling direct translation to a surprise metric without mathematical inversion or negation. After observing the result $m_{t}$ of the chosen action, the surprise $S_{t}\left(m_{t},a_{t}\right)$ is computed and then linearly combined with the causality term to guide the action‐selection in the next step:

(8) $$p\left(a_{t+1}\right)\propto C\left(m|a_{t}\right)+\alpha \cdot S\left(m_{t},a_{t}\right)\;,$$

where α is a hyperparameter that balances the relative importance of the CAIS term and the surprise term. Greater α values will increase the strength of the extinction burst in simulations. We note that α could be used to model individual differences between children.

## 3 Simulation Setup and Results

The simulations of the basic mobile paradigm comprise three sequential phases—baseline, connect, and disconnect. Time is discretized into steps of 0.5 s. An entire simulation takes 2000 time‐steps. Initially, the *baseline phase* runs for 200 time‐steps (100 s) to establish pre‐intervention behavioral frequencies. This is followed by the *connect phase* (1000 time‐steps, 500 s), during which one limb (randomly selected from four) is contingently linked to mobile motion, and concludes with the *disconnect phase* (800 time‐steps, 400 s), where all limb‐mobile contingencies are terminated. Crucially, causal relationships between limb movements and mobile activation are operationalized only in the connect phase, while the baseline and disconnect phases serve as pre‐ and post‐intervention controls.

In our computational model of the mobile paradigm, infants' independent limb control is simulated via k discrete action variables $a^{k}$ each corresponding to a limb and constrained to binary states (0: stationary, 1: moving). The mobile's motion m is modelled as a stochastic outcome variable. During baseline and disconnect phases, m is sampled from a baseline Gaussian distribution $N\left(0,0.3\right)$, permitting small random movements of the mobile reflecting environmental stochasticity. In the connect phase, however, m becomes contingent on the selected limb's action: if the agent activates the causally linked limb, m is sampled from a predefined normal distribution $N\left(5,0.3\right)$, simulating the mobile's movement; if the limb is inactive, m reverts to the noisy baseline. This design isolates causal learning to the connect phase while maintaining ecological validity through phase‐specific outcome dynamics.

To compute the CAIS, we independently evaluate each limb's causal impact by modelling the mobile outcome m as a conditional distribution dependent on the binary action state (0: stationary, 1: moving) of each limb k. Conditional distributions for each limb k are updated only when action $a^{k}$ is executed, ensuring that at each time step, only one conditional distribution per limb—corresponding to the selected action—is revised. Concurrently, the unconditional outcome distribution, estimated as a marginal distribution irrespective of actions, is updated at every time step to reflect the overall environmental outcome dynamics. Across all mobile‐paradigm simulations, we estimate these nine distributions (eight conditional and one marginal) via quantile regression, bypassing parametric density assumptions while directly modelling outcome percentiles to compute causal and surprise metrics. Quantile values (spanning 49 quantiles from 2% to 98%) are modelled as learnable parameters without any network, optimized via back‐propagation (Adam optimizer; Kingma and Ba 2015) with a loss defined in Equation (5) and a 1‐cycle cosine annealing learning schedule (Loshchilov and Hutter 2017) that smoothly decays the learning rate from 0.03 to 0 over the course of the simulation. This annealing strategy serves a dual purpose: its smooth decay curve provides a computational analogue for an infant's diminishing exploratory engagement over time, while the resulting low learning rate in later stages ensures model stability, allowing for a gradual and plausible decay of learned behaviors during the extinction phase. See Appendix A for the learning rate curve and the result without the cosine annealing strategy.

At each time‐step t, after updating the model and computing CAIS for every action‐state pair of each limb, we derive the limb‐specific causal engagement score by averaging CAIS values over both action states for each limb as $\bar{C_{t}}\left(m|a^{k}\right)$. To prevent perpetual action activation and maintain exploratory behavior, we introduce a baseline causal inertia term for each limb, computed as the mean CAIS $\bar{C_{t}}\left(m\right)$ across all state‐action pairs. This inertia represents the default causal “cost” of inaction, ensuring the agent does not overcommit to movement without evidence of contingent outcomes. For action selection, each limb's causal engagement score is normalized against its baseline inertia using a sigmoid function to derive the corresponding movement probability:

(9) $$p\left(a_{t+1}^{k}=1\right)=\frac{e^{\bar{C_{t}}\left(m|a^{k}\right)}}{e^{\bar{C_{t}}\left(m|a^{k}\right)}+e^{\bar{C_{t}}\left(m\right)}}=\frac{1}{1+e^{\bar{C_{t}}\left(m\right)-\bar{C_{t}}\left(m|a^{k}\right)}}$$

This normalization ensures that limbs with higher causal influence scores (relative to the limb's baseline causal inertia) are prioritized for activation, dynamically aligning movement frequency with inferred causal efficacy while maintaining exploratory behavior through stochastic action selection.

**FIGURE 1:**
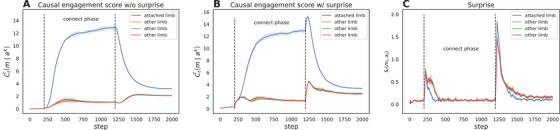
Causal engagement scores in the mobile paradigm simulation. **Panel A**: causal engagement score without *surprise*. **Panel B**: causal engagement score with *surprise*. **Panel C**: The *surprise* of each limb. Blue lines depict causal engagement scores for contingently connected limbs; other lines represent non‐contingent limbs. Dashed vertical lines mark phase transitions (baseline → connect → disconnect). Shaded areas indicate ±1 standard deviation across 5 simulation runs.

Results of each limb's causal engagement score $\bar{C_{t}}\left(m|a^{k}\right)$ in an example simulation are shown in Figure 1. Panel A illustrates the behavior of the model without the surprise mechanism. We observe a marked rise in movement probability for the causally connected limb during the connect phase, reflecting the model's adaptation to learned action‐outcome contingencies. Non‐connected limbs exhibit a modest, transient increase in activation during this phase—a by‐product of spurious correlations arising from stochastic environmental feedback, which the model initially misattributes to causal efficacy. Following the disconnect phase, the previously connected limb's movement probability declines sharply, while non‐connected limbs experience a brief resurgence in activation due to the normalization mechanism (Equation 9) redistributing behavioral priority across all limbs. This pattern mirrors exploratory hypothesis‐testing behavior, where the agent temporarily shifts focus to alternative limbs to probe for new causal relationships. Over time, movement probabilities converge to a stable low baseline, stabilized by a cosine annealing scheduler that gradually decays the exploration rate—a computational analogue to behavioral “exhaustion” observed in infants, where prolonged environmental disengagement reduces novelty‐seeking motivation. The non‐zero convergence reflects persistent stochasticity in action‐selection, ensuring continuous sensitivity to potential contingencies.

To model extinction bursts, we first compute the action‐specific surprise for each limb k at time‐step t using Equation 7, which quantifies the mismatch between predicted and observed outcomes. The causal engagement score for each limb is then dynamically adjusted by replacing it with a linearly weighted combination of the original score and the surprise term, as defined in Equation 8. This adjustment propagates to the non‐movement activity rate, which is recalculated to reflect the updated causal engagement scores across all limbs. Action‐selection probabilities for the subsequent time‐step are derived via Equation 9, incorporating these modified scores. To stabilize the inherently stochastic surprise signal, each limb's adjusted causal engagement score is further smoothed using an exponential moving average, approximating the agent's gradual refinement of causal hypotheses in response to persistent expectation violations:

$$\bar{C_{t+1}}\left(m|a^{k}\right)=\varepsilon \cdot \bar{C_{t+1}}\left(m|a^{k}\right)+\left(1-\varepsilon \right)\cdot \bar{C_{t}}\left(m|a^{k}\right),t>1$$

with ε=0.03. This dual integration of surprise and causal inference captures extinction bursts as transient behavioral recalibration, where abrupt environmental discontinuities temporarily amplify limb‐specific exploration to resolve causal uncertainty.

In panel B of Figure 1, a transient extinction burst manifests as a surge in movement probability for the previously connected limb at the onset of the disconnect phase, contrasting with the immediate gradual decline in A. This spike reflects a prediction error—a quantifiable violation of the agent's learned causal model, wherein the limb's movement no longer elicits the anticipated mobile motion. Such deviation between expected (causally inferred) and observed outcomes triggers a compensatory behavioral response: the infant model intensifies actions associated with the now‐disrupted contingency, attempting to restore the violated causal hypothesis. This aligns with neurodevelopmental accounts of extinction bursts as active hypothesis‐testing under uncertainty, where agents transiently amplify exploratory behaviors to resolve environmental incongruities. This process is driven by the underlying surprise metric shown in Panel C. Here, surprise for all limbs is initially high, reflecting the model's uncertainty. During the connect phase, surprise decreases as the model learns the environment's dynamics, with a markedly faster decay for the attached limb as its outcomes become highly predictable. At the onset of the disconnect phase, the absence of the expected mobile motion creates a massive prediction error, causing a dramatic and immediate spike in surprise, which is more pronounced for the previously attached limb whose causal model has been most directly violated.

**FIGURE 2:**
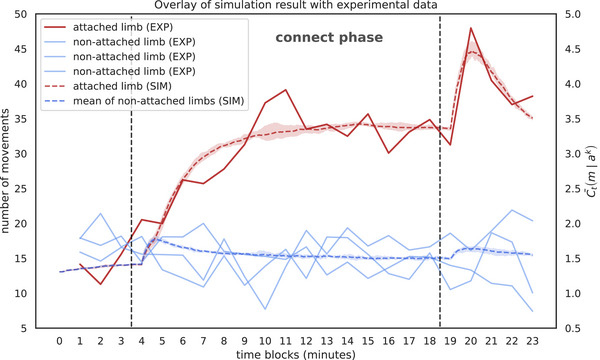
Overlay of simulation result with experimental data from Rovee‐Collier et al. (1978). Temporal scaling: 100 simulation steps = 1 min. Experimental results (solid lines) and simulation outputs (dashed lines) for causal engagement scores are shown. Red denotes the contingently connected limb; blue represents simulated non‐contingent limbs. Shaded areas indicate ±1 standard deviation across 5 simulation runs.

To validate our model against empirical infant behavior, we systematically varied the surprise weighting parameter α—which balances causal inference against novelty‐driven exploration—and scaled response magnitudes to align with observed movement frequencies in infant studies. We also slightly adjusted the duration of each time step (100 time steps corresponding to 1 min instead of 50 s). As shown in Figure 2, simulations with α=10 (optimized via grid search) yield causal engagement scores that closely match experimentally measured limb‐specific movement rates during extinction bursts (Rovee‐Collier et al. 1978). This alignment demonstrates that our framework captures the non‐linear amplification of limb activation following contingency removal, a hallmark of infant extinction bursts. By grounding parameter selection in empirical data, we bridge computational theory with developmental phenomenology, positioning our model as both a replicative and predictive tool for studying early causal cognition.

### 3.1 An Inverted Case

To benchmark our causal model against pre‐wired associative learning frameworks, we implemented a naïve direct reinforcement agent that replicates the “babybot” simulation. This agent operates on a principle of direct operant conditioning, modulating limb movement probabilities without forming an explicit causal representation of its environment. The model is tabular in nature, directly maintaining and updating a probability of movement for each of the four limbs. These probabilities are initialized to a uniform value of 0.2. The agent's learning is driven by the temporal co‐occurrence of its actions and environmental feedback. Specifically, when a limb movement is followed by a significant mobile response‐simulated as a draw from a Gaussian distribution $N\left(5,0.3\right)$ exceeding a threshold of 2.5‐the corresponding movement probability for that limb is increased by 1%. Conversely, if a limb movement is not followed by a significant mobile response, the probability is decreased by 0.5%, effectively punishing inaction or ineffective action. The architecture of this model is intentionally simplistic to provide a clear and falsifiable instantiation of the pre‐wired associative learning hypothesis. The model is further characterized as “naïve” because its learning mechanism is purely correlational. It is designed to detect and exploit temporal contiguity between an action and a subsequent “reward,” but it possesses no mechanism to distinguish whether this correlation is the result of a genuine causal link or a mere statistical coincidence. The hard‐coded threshold of 2.5 on the mobile's movement serves as a predefined, binary reward signal. The model's entire operational logic is to increase the frequency of any action that is reliably followed by this signal. In doing so, it bypasses the fundamental cognitive challenge of identifying a true contingency from a vast space of possibilities (Gergely and Watson, 2014). It is not discovering a causal relationship; it is merely optimizing its behavior to maximize the frequency of a desired outcome.

Crucially, this direct reinforcement agent lacks causal reasoning, instead relying on action‐outcome correlations. To further validate the robustness of our causal model, we conducted an inverted simulation: the mobile moves by itself but halts when the connected limb is active. This inversion tests whether the model can infer non‐intuitive causal relationships (e.g., “my action stops the mobile”) rather than defaulting to heuristics like “movement causes motion.”

**FIGURE 3:**
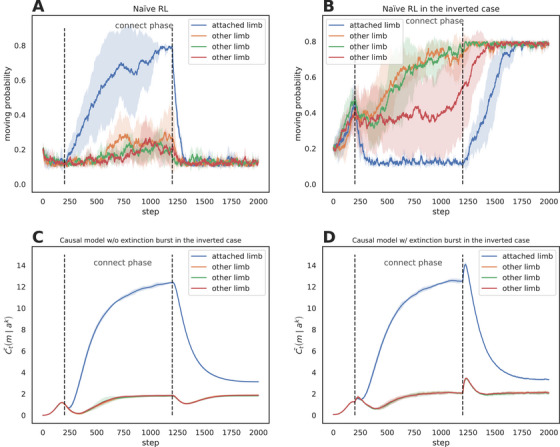
Model performance in standard and inverted paradigms. **Panel A**: Naïve reinforcement (standard). **Panel B**: Naïve reinforcement (inverted). **Panel C**: Causal model (inverted, no extinction burst). **Panel D**: Causal model (inverted, with extinction burst). Shaded areas indicate ±1 standard deviation across 5 simulation runs.

As illustrated in panel A of Figure 3, the direct reinforcement model successfully replicates increased movement probability for the causally linked limb during the connect phase in the standard mobile paradigm, where mobile motion serves as a pre‐specified reward signal. However, in the inverted simulation (panel B)—where limb activation suppresses mobile motion—the reinforcement model increases the movement frequency of unconnected limbs while suppressing that of the connected limb. This divergence arises because the direct reinforcement framework rigidly equates action‐reward correlations with causal efficacy, lacking the capacity to invert learned associations. In contrast, the causal model (panels C and D) dynamically infers latent causal structures by identifying motion suppression as the operative causal mechanism, and—facilitated by the surprise module—triggering an extinction burst when suppression fails during the disconnect phase. By decoupling causal discovery from pre‐specified rewards, the model generalizes across arbitrary action‐effect relationships, demonstrating its robustness to environmental reversals. The qualitatively different predictions of the direct reinforcement versus the causal model for the inverted case make it an interesting experimental paradigm to explore in future studies.

### 3.2 An Alternative Controllability‐Based Model

Controllability models are widely used to detect action influence (Touchette and Lloyd 2004). However, they exhibit a systematic bias toward inferring influence only when actions reduce outcome entropy (i.e., stabilize predictable states). This framework fails when an action increases environmental uncertainty—for instance, randomizing mobile motion by shifting it from deterministic to stochastic dynamics—as such scenarios violate the assumption that controllability necessitates low‐entropy outcomes. In contrast, our causal model, grounded in distributional difference identification, dynamically infers influence irrespective of entropy shifts, identifying actions as causal even when they amplify variability, thereby generalizing beyond stability‐centric heuristics.

To empirically assess controllability models' entropy bias, we constructed a controllability‐based variant of our framework by replacing Equation 3 with a controllability heuristic:

$$C\left(m|a\right)=\frac{1}{\left|A\right|}\sum_{a\in A}\left(H\left(m\right)-H\left(m|a\right)\right)\;,$$

where A is the support of the action space and H is the entropy of outcome m, which is derived through differential quantile entropy (Vasicek 1976):

$$H\left(q\right)=\int_{0}^{1}\log q^{\prime }\left(\tau \right)d\tau \;,$$

where $q^{\prime }\left(\tau \right)$ is the derivative of the quantile function q(τ). For simulation, to challenge the causal model's capacity to detect non‐obvious action‐outcome relationships, we replaced the original unimodal outcome distribution $N\left(5,0.3\right)$ with a bimodal Gaussian mixture comprising two equally weighted components: $N\left(-2,1\right)$ and $N\left(2,1\right)$. Unlike controllability models, which conflate causal influence with entropy reduction, our framework successfully identifies the causal link between limb movements and the emergence of bi‐modality—despite the outcome distribution's increased entropy. This demonstrates the model's ability to infer causality through distributional restructuring rather than stability‐seeking heuristics, generalizing to scenarios where actions induce complex, multimodal outcome dynamics.

**FIGURE 4:**
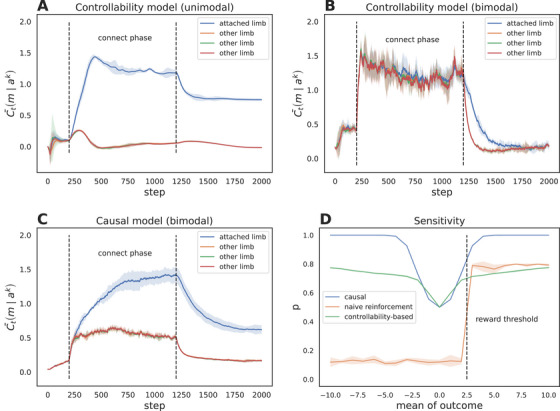
Comparison of controllability‐based model and causal model. **Panel A**: controllability model with unimodal outcome; **Panel B**: controllability model with bimodal outcome; **Panel C**: causal model with bimodal outcome. **Panel D**: Sensitivity to varying mean of outcome distribution in the unimodal case. Lines represent the moving probability of the contingent limb at the final time‐step of the connect phase for different models. The non‐movement outcome distribution is centered at 0. Shaded areas indicate ±1 standard deviation across 5 simulation runs.

As demonstrated in Figure 4, the controllability model successfully identifies causal influence in the unimodal paradigm (Panel A), where actions stabilize outcomes around a single mean. However, it fails to detect causal relationships in the bimodal case (Panel B), where limb movements shift outcomes to a high‐entropy mixture, reflecting its inherent bias toward equating controllability with entropy reduction. In contrast, our causal model (Panel C) robustly infers causal influence in the bimodal setting by quantifying distributional divergence between interventional and observational outcomes, independent of entropy constraints. This divergence highlights the limitations of stability‐centric frameworks for learning in complex, dynamic environments.

To evaluate model robustness against environmental variability, we systematically varied the mean of the outcome distribution in the unimodal case while holding variance constant, testing sensitivity across the causal, controllability, and direct reinforcement frameworks. We record the moving probability of the contingent limb at the final time‐step of the connect phase, quantifying how inferred causal efficacy scales with environmental reward magnitude. As shown in Figure 4, Panel D, the causal model maintains consistent causal inference accuracy across parametric shifts, whereas the controllability model exhibits sensitivity to mean proximity (e.g., failing when action‐contingent outcomes marginally exceed baseline). The reinforcement model, however, demonstrates catastrophic exploration collapse: when action‐contingent outcomes fall below or near the baseline outcome mean, it suppresses limb movements entirely, mistaking environmental stochasticity for non‐contingency. This divergence arises because the causal model's reliance on distributional divergence (e.g., Wasserstein distance) decouples learning from absolute reward magnitude, enabling robust discovery of agency in both high‐ and low‐reward regimes. These results underscore the limitations of reward‐maximizing and stability‐centric models in dynamic environments, while highlighting causal abstraction as a critical enabler of adaptive, open‐ended learning.

### 3.3 Simulation of Infants' Contingent Vocalization

To demonstrate the generality of our approach beyond the mobile paradigm, we simulated a recent infant vocalization experiment (Venditti et al. 2025), where 64 infants (age range: 7 months, 8 days to 8 months, 2 days) progress through four phases: baseline 1 (partial feedback), social response (contingent feedback), extinction (no feedback) and baseline 2 (partial feedback). Vocalizations were modelled as binary actions (0: silent, 1: vocalizing), with 50 simulation steps approximating 1 min of real‐time interaction. During the social response phase, vocalizations triggered contingent feedback (e.g., caregiver responses or remote‐controlled car activation), while the extinction phase abruptly terminated all feedback. Causal engagement scores—calculated as the divergence between vocalization‐contingent and baseline outcome distributions—were averaged per phase and normalized to match empirical vocalization rates. Because the baseline response rate was set to match each infant's caregiver's rate during the familiarization phase, specific empirical baseline data were not available. Therefore, we assumed a baseline value of 0.7 and restricted model validation to non‐baseline stages. (social response and extinction), aligning simulated vocalization rates with observed infant behavior in these phases.

**FIGURE 5:**
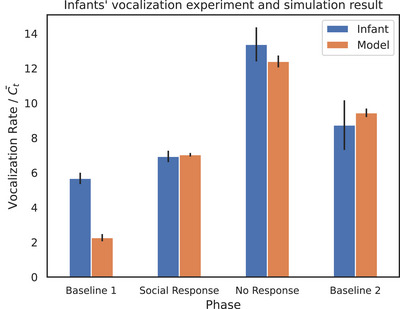
Simulation result of infants' vocalization data. Blue bars: experimental data (Venditti et al. 2025) (mean ±1 SD across trials). Orange bars: simulation outputs (mean ±1 SD across 5 runs). Simulation results are averaged in each learning phase. Temporal scaling: 50 simulation steps = 1 min.

As shown in Figure 5, the model replicates the empirical pattern: vocalizations rise during the social response phase (reflecting causal learning) and escalate further in the extinction phase, mirroring the extinction burst observed in infants. This surge aligns with our prediction‐error hypothesis, where terminated contingencies trigger compensatory attempts to restore expected social feedback, demonstrating the framework's ability to generalize to the social‐cognitive domain.

## 4 Discussion

While the concept of causality is central to studies of infant motivation, which have predominantly focused on constructs like control and agency (Gopnik and Schulz 2004; Watanabe and Taga 2011; Watson 1966), the specific computational mechanisms that enable infants to discover causal structure in open‐ended environments remain a critical area of investigation. Many existing computational accounts either bypass the discovery problem by relying on pre‐specified reward functions, as in simple reinforcement, or adopt heuristics, such as entropy reduction, that may fail in complex and more ecologically valid scenarios. Control, while closely related to causality, comprises two facets: (1) impact magnitude—quantifying how actions alter outcomes—and (2) predictive regularity—assessing how reliably actions map to outcomes. These dimensions align with information‐theoretic definitions of controllability, operationalized through marginal and conditional entropy metrics (Touchette and Lloyd 2004), which measure distributional differences between outcomes under intervention (e.g., limb movement) and baseline (inaction). While prior work employs measures such as the KL divergence to quantify such differences (Seitzer et al. 2021), our framework adopts a Wasserstein distance paired with quantile regression (see Section 3), offering advantages in terms of symmetry, computational efficiency, and non‐parametric flexibility. Unlike entropy‐based methods, our approach bypasses integration over all possible interventions by independently modelling marginal outcome distributions—a design inspired by randomized controlled trials (RCTs) (Lee et al. 2019) and natural direct effect estimation (VanderWeele and VanderWeele 2015). Furthermore, while agency research emphasizes mediating factors (e.g., distinguishing direct vs. indirect control), our causality‐centric metrics provide a foundational lens for future studies to dissect how infants attribute causal efficacy to their own actions versus external agents (Sloan et al. 2023). By bridging controllability's quantitative rigor with causal abstraction, this work advances a unified framework for understanding how infants discover and adapt their influence over dynamic environments.

The extinction burst phenomenon, while its underlying motivation remains debated, aligns with expectation‐violation accounts (discussed in Section 1), wherein infants amplify actions to resolve disrupted causal predictions. This explanation contrasts with controllability‐based models (Section 3.2), which inherently penalize actions that induce high outcome uncertainty, as they prioritize stable, low‐entropy states. Similarly, traditional causal and controllability models implicitly disincentivize uncertainty by optimizing for deterministic action‐outcome mappings, creating an exploration‐exploitation tension: agents must balance exploiting learned causal relationships with exploring novel hypotheses. Our model addresses this tension by linearly weighting expectation violation (exploration) against causal certainty (exploitation). We chose this simple heuristic as the real neuro‐cognitive mechanisms governing this trade‐off in infants are unresolved. By decoupling exploitation (causal inference) and exploration (surprise‐driven hypothesis testing) into modular components, future work could systematically investigate how developmental stages modulate this balance—for instance, whether infants prioritize exploration during early learning phases before shifting to exploitation as causal models stabilize (Gopnik et al. 2017, 2020). This framework offers a pathway to unify behavioral observations with computational principles of adaptive learning across development. Interestingly, (Spisak et al. 2025) developed a framework that successfully models limb‐specific activity increases and extinction bursts‐particularly in baseline versus control and binary versus non‐binary groups‐without explicitly modelling agency or surprise. Instead, they utilized specialized computational schemes to derive loss functions that encourage a trade‐off between predictability and novelty. However, the reliance on a hard‐coded novelty threshold and MSE‐based prediction error limits the model's ability to handle complex stochastic outcomes, such as the bimodal case discussed in our simulations (Section 3.2). Furthermore, because designing loss functions is conceptually similar to reward engineering, this approach may not constitute a distinct alternative to reinforcement learning.

This work is subject to several limitations. First, the proposed causality‐based contingency detection framework serves primarily as a proof‐of‐concept. Consequently, the simulation simplifies the infant experience: complex motor movements are reduced to ballistic binary actions, and high‐dimensional visual inputs are represented by numerical samples of a scalar random variable representing the amount of mobile movement. Second, the model's predictions regarding the Inverted and Bimodal cases (Sections 3.1 3.2) require validation through empirical experiments to confirm their relevance to infants' sense of agency. Specifically, if our model's predictions are confirmed, infants in the Inverted and Bimodal cases would still amplify the movement frequency of the contingent limb relative to the other limbs. A further limitation is the absence of a non‐contingent control condition characterized by random or experimenter‐initiated mobile movement (Angulo‐Kinzler et al. 2002; Popescu et al. 2021; Sloan et al. 2023). Addressing these constraints is left for future research.

This work lays the foundation for several promising avenues in computational modelling of cognitive development. First, extending the framework to multimodal learning—integrating proprioceptive, haptic, and auditory feedback—could capture how infants generalize causal agency across sensory domains, such as linking vocalizations to caregiver responses or object manipulation to tactile outcomes. Addressing such questions has become feasible through simulation platforms such as MIMo, the Multimodal Infant Model (Mattern et al. 2022, 2024). Second, incorporating hierarchical causal reasoning would enable agents to infer higher‐order contingencies (e.g., contextual rules governing when actions yield effects), mirroring infants' ability to abstract causal schemas from sparse interactions. Third, coupling the model with neuroimaging (e.g., infant EEG/fNIRS) could map computational signals like prediction errors or Wasserstein distances to neural correlates (e.g., MMN, theta oscillations), bridging computational and biological levels of analysis. Additionally, embedding developmental trajectories—simulating maturational changes in exploration‐exploitation balance—could elucidate how causal learning strategies evolve from infancy to adulthood.

Finally, deploying these models in physical robots could test their utility in the engineering domain, fostering adaptive AI that, like infants, learns through curiosity‐driven experimentation in the physical world (Poli et al. 2024). By uniting causal inference, developmental psychology, and computational neuroscience, this framework offers a novel perspective for studying the origins of human agency and its lifelong plasticity.

## Conflicts of Interest

None of the authors have a conflict of interest to disclose

## Figures and Tables

**FIGURE 6:**
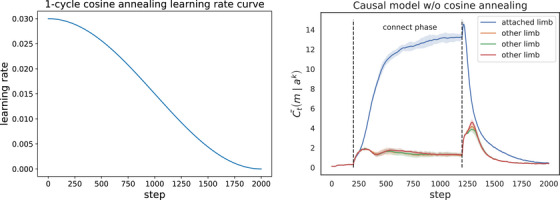
**Left**: The learning rate curve under the schedule of a 1‐cycle cosine annealing strategy. The learning rate decays from 0.03 to 0 across the simulation. **Right**: The causal engagement score with a fixed learning rate (0.03).

**FIGURE 7:**
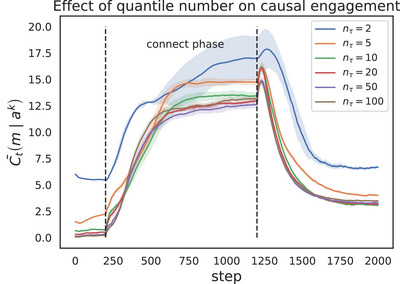
Causal engagement scores for the contingent limb across varying numbers of quantiles. Solid lines represent the mean, and shaded areas indicate ±1 standard deviation across 5 independent simulation runs.

## Data Availability

All data utilized in this study were sourced from publicly available published sources. The source code for data modeling will be made publicly available upon acceptance. None of the authors have a conflict of interest to disclose. We comply with Wiley's Research Integrity and Publishing Ethics. The source code for the computational models, along with the scripts used to run the simulations, can be accessed at TrieschLab‐CAIS.

## Appendix A

### A.1 Cosine Annealing Strategy

Figure 6 illustrates the learning rate curve for the 1‐cycle cosine annealing strategy (left) and the results using a fixed learning rate of 0.03 (right). Compared to Figure 1, the extinction burst is more pronounced in the unconnected limbs than in the connected ones. This is attributed to the constant high learning rate, which causes the learned quantile values to adapt to the new outcome distribution too rapidly.

### A.2 Sensitivity to the Number of Quantiles

To examine how quantile granularity affects modelling results, we varied the number of quantiles within our standard mobile‐conjugate paradigm simulation. We conducted simulations for $n_{\tau }\in \left\{2,5,10,20,50,100\right\}$; the resulting causal engagement scores for the contingent limb are presented in Figure 7. An increase in activity during the connection phase and a subsequent extinction burst were observed across all values of $n_{\tau }$ (corresponding to percentile intervals ranging from approximately 33% to 1%). However, lower quantile counts (e.g., $n_{\tau }=2$) resulted in high variability and jagged curves. Consequently, for the primary analysis presented in the main text, we selected $n_{\tau }=49$ (a 2% percentile interval) to balance curve smoothness with computational efficiency.
